# Optical Thin Films in Space Environment: Investigation of Proton Irradiation Damage

**DOI:** 10.1021/acsami.4c03362

**Published:** 2024-07-09

**Authors:** Alain J. Corso, Marta Padovani, Giovanni Santi, René Hübner, Ulrich Kentsch, Marco Bazzan, Maria G. Pelizzo

**Affiliations:** † Consiglio Nazionale delle Ricerche—Istituto di Fotonica e Nanotecnologie (CNR-IFN), Via Trasea, 7, 35131 Padova, Italy; ‡ Dipartimento di Ingegneria dell’Informazione, Università di Padova, Via Gradenigo 6B, 35131 Padova, Italy; ¶ Centro di Ateneo di Studi e Attività Spaziali (CISAS), Università di Padova, Via Venezia, 15, 35131 Padova, Italy; § Institute of Ion Beam Physics and Materials Research, Helmholtz-Zentrum Dresden-Rossendorf, Bautzner Landstraße 400, 01328 Dresden, Germany; ∥ Dipartimento di Fisica e Astronomia, Università di Padova, Via Marzolo 8, 35131 Padova, Italy

**Keywords:** coatings, metals, dielectrics, proton irradiation, space environment, optical instruments, telescopes

## Abstract

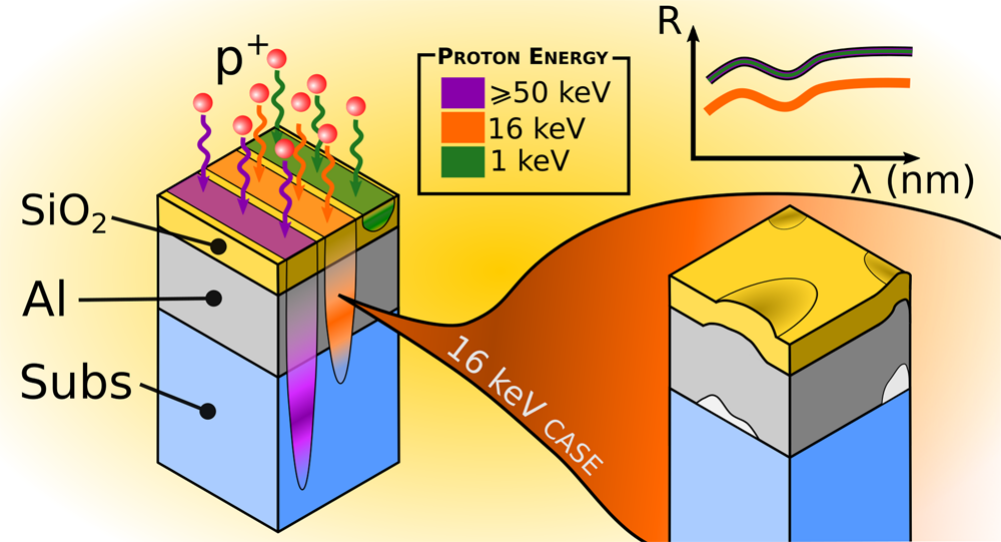

The present work reports a systematic study of the potential degradation of metals and dielectric thin films in different space environments. The mono- and bilayers selected are made of materials commonly used for the realization of optical components, such as reflective mirrors or building blocks of interferential filters. More than 400 samples were fabricated and irradiated with protons at different energies on ground-based facilities. The fluences were selected as a result of simulations of the doses delivered within a long-term space mission considering different orbits (Sun close, Jovian, and Geostationary orbits). In order to stress the samples at different depths and layer interfaces, experiments were carried out with a range of proton energies within 1 and 10 MeV values. An estimate of a safe maximum fluence has been provided for each type of sample at each energy. The damage mechanism, when present, has been investigated with different optical and structural techniques.

## Introduction

The exploration of the solar system and the exploitation of the resources present in extraterrestrial habitats are major scientific and technological goals of the coming years. This will involve a further expansion of the number of satellites in Earth’s orbit, as well as a desirable increase in space missions aimed at the exploration of planets and their satellites. This will require a greater understanding of the circumterrestrial environment and awareness of the effect it can induce on spacecrafts and instrumentation, as well as a deeper knowledge of space hostile environments.^1^ Some of the space missions operate in extremely hostile environments, such as the ESA Solar Orbiter mission (SolO),^2^ which will suffer high thermal gradients as well as high particle fluxes because of the sun-close distance, and the ESA Jupiter Icy Moons Explorer (JUICE), which will experience the high activity of the Jovian magnetosphere and its synergy with the Jupiter satellites.^3^ The impact of the space environment on satellites and probes directly depends on solar activity, and it is, therefore, essential to acquire scientific knowledge both in the field of space weather and in the interaction between particles and components/systems.^4,5^ Over the years, much attention has been given to electronic components, which are very sensitive to the presence of cosmic rays traveling throughout the whole heliosphere. However, the spacecraft components also need to be qualified for ions and electrons, as they are typically trapped in the magnetospheres. MeV protons and electrons are abundant, for example, in low-earth-orbit (LEO), because they are trapped in the Van Allen belts,^6^ while protons of low energy have also been demonstrated to be present in planetary atmospheres, including terrestrial orbits.^7^ High-energy protons are associated with eruptive phenomena such as coronal mass ejections and solar flares, while the quiet solar wind is associated with the presence of particles with lower kinetic energy, typically 1 keV for protons. Moreover, protons with energies in the range of 100 keV can be found in the near-Earth space environment.^8^ The use of simulators, which combine predictive models and experimental observations, allows us to describe the terrestrial environment in detail.^9,10^ The proton energy spectra highlight that low-energy particles are characterized by higher fluxes so that the fluences delivered at the end of missions are orders of magnitude higher than those associated with high-energy particles in the range of MeVs. While low-energy particles do not affect shielded components and systems, they can be detrimental to unprotected components directly exposed to radiation, such as mirrors, filters, and windows. For this reason, the study of the damage induced by low-energy particles on optical materials, thin films, and coatings in the space environment is pivotal for the realization and optimization of scientific instrumentation, navigation sensors, and solar panels.^11−15^

The present paper aims to report the results obtained within the ESA GSTP Project No. 4000122836/18/NL/PS/gp entitled *Radiation Testing of Optical Coatings for Space*. For the first time, a selection of thin-film coatings largely employed in space instrumentation have been irradiated with low- and high-energy protons, helium ions, and electrons by following a systematic approach. One of the main purposes of the project was to provide the scientific community with a guide to choose the materials that better withstand the space environment foreseen for their operation but above all to guide it in choosing the test parameters for components validation. For instance, it has been already demonstrated that proper selection of the proton energy is fundamental for the evaluation of the stability of the interfaces of a thin-film structure,^16^ while a first set of recommendations for the testing of coated components with ground-based accelerator facilities have been already provided;^17^ in particular, high particles rates should be avoided, as they can induce thermal processes not present in space, where the exposure often lasts years. Within this work, it will be demonstrated how experimental parameters such as flux and energy should be carefully selected based on the sample materials and structure design, avoiding simply referring to practical reasons, such as the availability of accelerators. Moreover, fluence curves for various space environments will be provided.

Within this work, over 400 samples were subjected to proton irradiation to assess their capacity to withstand such damage agents. Each type of sample was irradiated with beams at different energies, fluxes, and fluences to investigate the dependence of the damage from each of the irradiation parameters. The proton energies used in the experimental sessions were the following: 1 keV, 16 keV, 50 keV, 100 keV, 1 MeV, and 10 MeV. The coatings under analysis were single layers and bilayers. Common metal and dielectric layers were considered, such as Au, Al, SiO_2_, TiO_2_, and ZrO_2_. Single-layer coatings were used as proof of the damage experienced by the material itself, whereas bilayers were used to test the shielding capabilities of some dielectric thin-film coatings as well as the irradiation-related effects at the interfaces between the materials. Due to their limited thickness (between a few and hundreds of nanometers), single layers are affected by protons of a few keVs of energy, as these implant inside the film, while higher-energy particles overcome the coating and reach the substrate. To test the irradiation at the interface between layers, the top-layer thickness was opportunely sized to place the peak of implantation of 16 keV protons at the interface between the first and the second layer. The results confirmed that the induced physical effects are finely energy-dependent.^16^ Dielectric bilayers such as TiO_2_/SiO_2_ and ZrO_2_/SiO_2_ have also been considered as they are building blocks of more complex structures such as interference filters. SiO_2_ single layers were deposited on sapphire to enhance its optical contrast with respect to the substrate material, while the remaining transmitting coatings were deposited on Suprasil.

The experimental results were used to determine the guard threshold fluence for each type of coating when experiencing some damage following proton irradiation. It is well-known that for fluences above of 10^17^ cm^–2^, low-energy ions implanting in metals, such as W, Au, and Cu, can result in bubble formation.^17−20^ A sponge-like morphology was observed in the case of gold films, associated with bubbles whose size increases with the fluence and forming large blisters for fluences of the order of 5 × 10^17^ cm^–2^.^17,21^ Various experiments of H- and He-ion irradiation on metal thin-film multilayers used in nuclear physics were carried out,^22−24^ as well as for components used in lithographic apparatus.^25−28^ The irradiation of charged particles can induce charge accumulation and alter the optical performance of extreme-ultraviolet multilayers via changes in the surface morphology and optical and structural characteristics, which, in the worst cases, can result in major damages, such as delamination and blistering.^29−32^ More data come from recent experiments that test Al and Ag metallic coatings for space applications^33^ and in particular with He-ion irradiation,^34^ which however do not define the damage thresholds for which the formation of these bubbles determines a degradation of the optical properties. In particular, common metal coatings, such as Al and Au thin films, have not been systematically studied so far.

The information on dielectric material thin films is very limited, as only a few experiments of low-energy ion irradiation on optical coatings have been systematically performed. Experiments with low-energy protons and fluences between 10^12^ cm^–2^ and 10^15^ cm^–2^ showed changes in the optical performance of various oxides,^35,36^ which were modeled in terms of refraction index changes. Some isolated experiments have been carried out on metal-protected bilayer optical coatings. For example, an SiO_2_/Al bilayer was irradiated with a 200 keV beam up to fluences of the order 10^17^ cm^–2^.^37^ The ion energy was selected in order to guarantee the implantation in the metals, overcoming the capping layer, showing again bubble formation. As already mentioned, a bilayer TiO_2_/Al coating was irradiated with three different ion energies to characterize the effect in the metal itself, in the dielectric protective layer, and at the interface between the two, showing that the damage induced depends on the energy and the design of the coating.^16^

The results obtained so far find their natural explanation provided by the systematic study carried out in this work. In the Operational Environments section, a description of some relevant environments is reported. In the Materials and Methods section, the optical coatings design, the irradiation test plan, and the sample characterization techniques are reported. Finally, in the Results and Discussion section, transmittance and reflectance curves were analyzed in order to provide, for each type of sample and irradiation session, a safety fluence, i.e., a maximum fluence that guarantees no degradation. Morphological and structural analyses (atomic force miscroscopy (AFM), transmission electron microscopy (TEM), and grazing incidence X-ray diffraction (GIXRD) were used to investigate the root causes of the damages observed.

## Operational Environments

The severity of the damage induced in thin films depends not only on the film material and its thickness but also on the energy, fluence, and flux of the particle beam. The differential flux corresponds to the number of particles incident on a surface per unit of area, time, and energy, and it is expressed in [N cm^–2^ s^–1^ MeV^–1^]. By integration over time, the differential fluence, in [N cm^–2^ MeV^–1^], can be retrieved. Otherwise, the flux can be expressed in integral form, in [N cm^–2^ s^–1^], which is obtained by integrating the number of particles of energies above a certain threshold. Also in this case, by integrating the flux with respect to time, we can obtain the fluence in the integral form. In the present work, the parameters of the irradiation sessions were defined according to the integrated values.

Various operating environments were considered, such as the geostationary earth orbit (GEO), the sun-close orbit, as in the case of the ESA Solar Orbiter (SolO), and the Jovian environment, such as in the case of the ESA Jupiter Icy Moons Explorer (JUICE) mission. In order to evaluate the associated charged particle parameters, simulations and models were combined. Simulations for protons revealed that the GEO orbit is the most challenging among the close-Earth operational environments, so the components that withstand it are qualified for LEO orbits. However, in the LEO orbit, additional simulations and tests are needed, as besides charged particles, high atomic oxygen is present with fluxes that depend on the orbit altitude. In GEO orbits, two sources of protons coexist: trapped particles, with energies below 1 MeV, and solar energetic particles (SEP) with energies reaching values up to hundreds of MeV. In the present work, the environment associated with a GEO mission lasting 15 years was considered. Regarding the fluence associated with the trapped protons in the Van Allen belts, this can be evaluated using both global and local models. Global models include NASA’s AP8 model (energy range 0.1–400 MeV)^38^ and Air Force Research Laboratory’s AP9 (energy range 1.15–164 keV and 0.1–400 MeV) models.^39^ Local models are IGP (energy range 0.1–38 keV and 80 keV to 300 MeV)^40^ and OPAL (energy range 80–800 MeV).^41^ The most powerful way to combine the results provided by the different models is using GREEN,^40^ implemented in the software OMERE. This “super” model can provide proton fluxes for different Earth’s magnetic field shells (i.e., shells with McIlwain *L*-parameters ranging from 1 to 8) at any local time, covering an energy range between 1 keV and 800 MeV for protons and between 1 keV and 10 MeV for electrons. GREEN uses both global and local models to calculate the trapped particle fluence by choosing the most suitable model for a given energy and position. On the contrary, SEP occurs when protons are accelerated from the sun surface through various eruptive phenomena, such as flares and coronal mass ejections. As for trapped particles, it is possible to compute the fluence of solar protons with various models. JPL-91 is a predictive model for proton fluences above the thresholds of 1, 4, 10, 30, and 60 MeV, and it is based on data collected by satellites within extended operational periods.^42^ ESP is used for providing cumulative fluences of solar protons with energies in the range 0.1 MeV to 1 GeV at 1 AU,^43^ combining experimental data and a physical model. SAPPHIRE combines past satellite data and a physical model to generate fluences in the range 0.1 MeV to 1 GeV using Monte Carlo simulations.^44^

**Figure 1:**
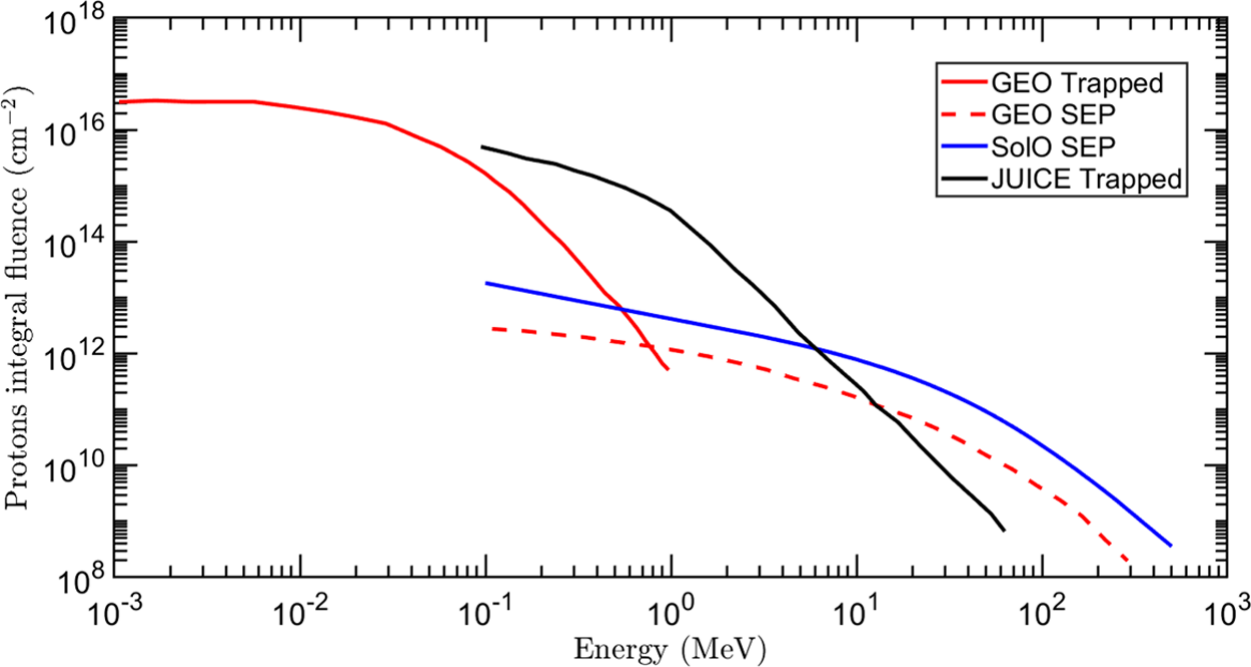
Integral fluences of protons for the different mission scenarios considered in this work.

Simulations for the present work were carried out using the OMERE software: GREEN was used for protons trapped in the radiation bands, as it combines AP8, SPM, and OPAL, while ESP was used for the solar protons. In Figure 1, the integral fluences of the solar and trapped protons corresponding to a 15-years mission exposure in the GEO orbit are shown. Up to 100 keV, the dominant contribution comes from trapped protons, while between 100 keV and 1 MeV, the total fluence is the sum of the output of the GREEN and ESP simulations. Above 1 MeV, only SEP are present.

For the space environment of a Sun-close mission, two contributions of particles have to be considered: the quiet solar wind and the SEP. In the case of the quiet solar wind, almost the whole flux of particles is composed of protons with an average energy of about 1 keV. Considering a typical mission life-span of 10 years with an average distance of 0.5 AU (i.e., ESA SolO mission), the total proton fluence given by the quiet solar wind is estimated to be ≃10^17^ cm^–2^.^12,30^ The SEP fluence was computed using the SPENVIS software,^45^ again for a 10-year mission at 0.5 AU Sun distance, by setting the launch date on February 2020. The simulation is reported in Figure 1.

To describe the JUICE mission environment, the ESA issue^46^ was used as a reference for the definition of the Jovian radiation environment at a final altitude of 200 km. Figure 1 shows the integral proton fluence during the Ganymede phase of the mission, where 60% of the total mission fluence is delivered. Solar protons were found to have a negligible effect at low altitudes, being mainly encountered during the interplanetary transfer phase.^46^ The dominant effect at 100 keV is therefore attributed to trapped protons, with fluence values of the same amount as those observed for a 15-year GEO mission.

## Materials and Methods

The materials and structures of the samples considered in this study, which include metallic and dielectric single layers, dielectric bilayers, and protected mirrors, are reported in Table 1. The selected coatings are commonly used in space applications to manipulate light in the near-ultraviolet (NUV, λ = 200–400 nm), visible (VIS, λ = 400–800 nm), and near-infrared (NIR, λ = 800–1300 nm) spectral ranges. For instance, dielectric layers such as SiO_2_, TiO_2_, and ZrO_2_ are the building blocks to realize antireflection coatings, interferential filters, and protective layers of metallic films, while the selected metals are largely exploited for the realization of mirrors in the UV–VIS range (Al), in the VIS–IR range (Ag), and in the IR (Au) range. The nominal structure parameters refer to those that were optimized according to the criteria reported below, while the deposited ones refer to those measured by profilometry or AFM. In the case of metallic coatings, Si wafers, while dielectric samples were deposited both on Suprasil and Si wafers. The Suprasil substrate is needed for transmittance characterization, whereas the thin-film structure on Si wafer is used for any structural characterization, in particular for TEM. Thus, each sample on Suprasil has its own equivalent on the Si wafer, deposited in the same run and irradiated in the same session. The only exception is SiO_2_, which was deposited on sapphire in place of Suprasil, in order to guarantee a high optical contrast between the film and the substrate.

**Table 1:** List of Samples

label	nominal structure	deposited structure	adhesion layer	substrate
S1W	Au (240 nm)	Au (195 nm ±1 nm)	Cr	Si wafer
S2W	Al (200 nm)	Al (210 nm ±1 nm)	Cr	Si wafer
S3GUV	SiO_2_ (520 nm)	SiO_2_ (512 nm ±1 nm)		sapphire
S4G/S4W	TiO_2_ (360 nm)	TiO_2_ (375 nm ±1 nm)		Suprasil/Si wafer
S5G/S5W	ZrO_2_ (340 nm)	ZrO_2_ (346 nm ±1 nm)		Suprasil/Si wafer
S6W	SiO_2_ (80 nm)/Al (200 nm)	SiO_2_ (78 nm ±1 nm)/Al (200 nm ±1 nm)	Cr	Si wafer
S7W	SiO_2_ (80 nm)/Ag (210 nm)		Cr	Si wafer
S8G/S8W	SiO_2_ (230 nm)/TiO_2_ (83 nm)	SiO_2_ (233 nm ±1 nm)/TiO_2_ (85 nm ±1 nm)		Suprasil/Si wafer
S9G/S9W	SiO_2_ (230 nm)/ZrO_2_ (104 nm)	SiO_2_ (230 nm ±1 nm)/ZrO_2_ (103 nm ±1 nm)		Suprasil/Si wafer

Single and bilayer coatings were fabricated. Single layers were used to understand the properties of the material itself, while bilayers are helpful to investigate also the stability of the film at the interfaces. The reflective bilayers are representative of the final components, while the transmitting ones, as said, are the elemental blocks of a repeating multilayer structure typical of a filter.

To minimize the occupation of the irradiation facility and thus the number of sessions, the proton energy was first selected (i.e., 1, 16, 50, 100, 1, and 10 MeV), and then the layer thickness of the various coatings was finely tuned in order to gain information on the stress-induced at the layer interfaces at those energies. In this way, different coating materials and structures can be tested together within the same session.

The layer thickness optimization was performed taking into account the following key facts and criteria.

(1) The 1 keV proton implantation distribution is narrow, with its peak close to the coating’s surface. In the case of bilayers or protected mirrors, the top layer is thick enough to guarantee that the implantation profile falls in the topmost part of it. Typical penetration depth values at this energy range vary from 40 to 70 nm. At this energy, the protective properties of the top layer materials are tested.
(2) 16 keV protons fully implant into the single-layer coatings, without reaching the substrate. In the case of the dielectric bilayers, the 16 keV protons have the implantation peak at the interface between the two layers, in order to stress the adhesion between them. In the protected metallic mirrors, the implantation peak falls in the middle of the metallic layer, stressing the whole coating structure.
(3) In the monolayers, the 50 keV proton implantation peak is at the interface between the coating and the substrate.
(4) Protons having energies higher than 100 keV pass through the whole coating and mainly implant into the substrate.

In order to size the thickness of the layers reported in Table 1, implantation profiles inside materials for the different energies were preliminary generated with the SRIM/TRIM software.^47^ Such simulations were performed considering the proton beam incidence angle that will be used in the irradiation experiments, which is 7°. It was also verified that the Cr adhesion layer (thickness of about 10–15 nm) can be ignored in simulations, as it does not affect the distribution of the backscattered ions due to its negligible quantity.

**Figure 2:**
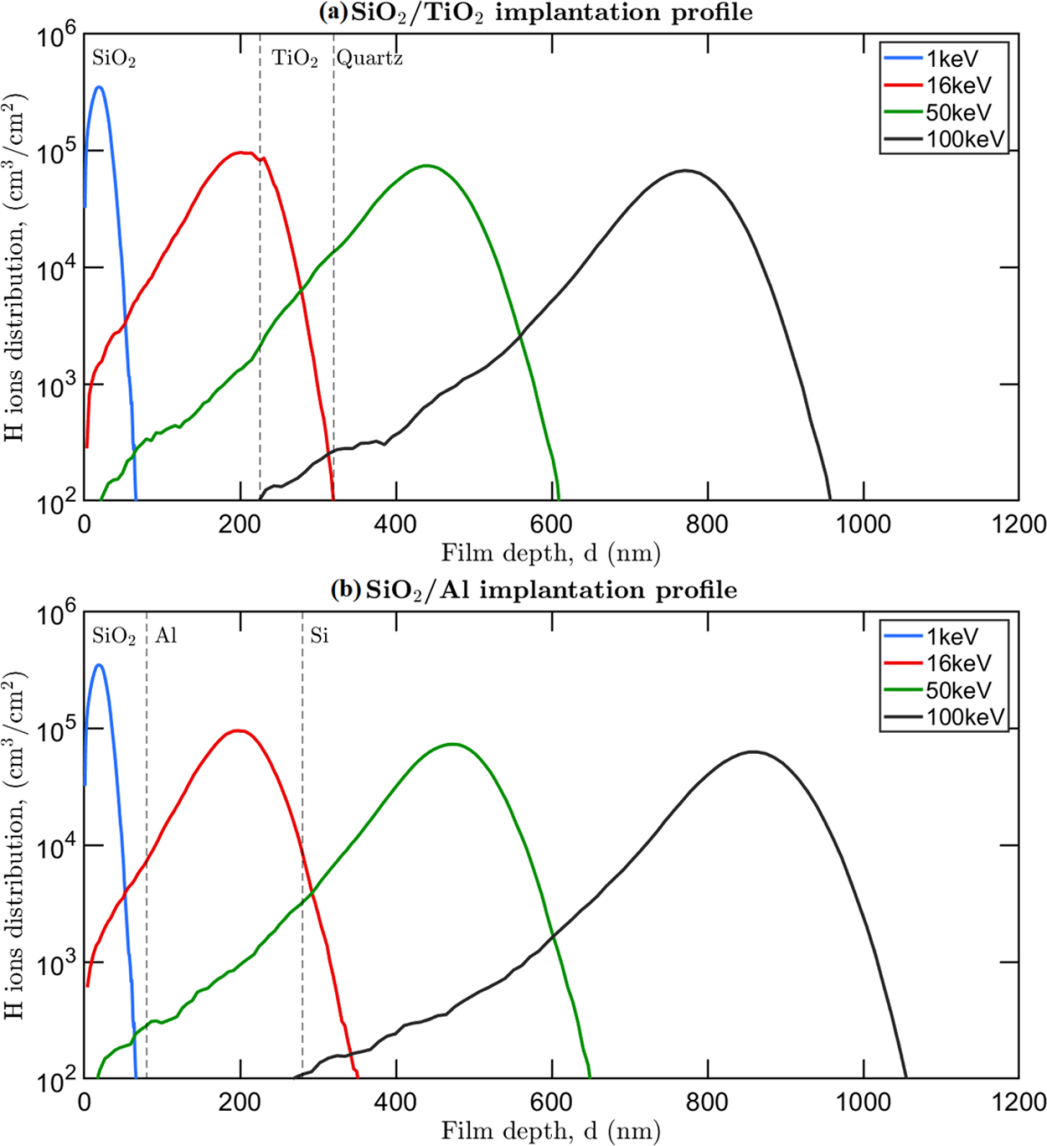
Proton implantation profile in an SiO_2_/TiO_2_ dielectric bilayer (sample S8G) (a) and in an SiO_2_-protected Al mirror (sample S6W) (b). The adhesion layer is neglected.

In the case of monolayers, the thickness of the layer was simply optimized to have the 50 keV protons peak placed at the substrate interface. In the case of the bilayers, simulations were first used to define the depth of the 16 keV proton peak. In the case of dielectrics, such depth sets the thickness of the top layer, optimized to guarantee that the proton peak is at the interface between the two materials; in the case of metals, this rule has not been applied, as it would have required a top layer with a too large thickness, departing from what was actually used. In dielectrics, the second layer thickness was sized wide enough to guarantee that most of the protons are implanted in it (according to simulations, 99.99% of protons are implanted in the coating); in metals, the second layer thickness was sized to have 16 keV protons peak at the center of the second layer itself. As a consequence, in both metals and dielectrics, the top layer thickness ensures that the 1 keV protons are fully implanted in it. As an example, the implantation profiles in samples S6W and S8G are reported in Figure 2a and Figure 2b, respectively.

Samples were prepared via e-beam evaporation deposition, eventually using ion beam assistance, adopting an in-line control for measuring the final performance; however, this method caused slight differences between the nominal and as-deposited thicknesses, which must be attributed to the difference between the actual film density and those used in simulations; the different density resulted in a change of the thickness of the layers with respect to nominal, necessary to achieve the same transmittance/reflectance of the target curve. The only coating for which the nominal and as-deposited structures differ significantly is the gold one (sample S1W); in this case, the sample thickness was limited by the fabrication process. All materials were deposited starting from pellet evaporation sources with purity better than 99.95% and placed in different crucibles, depending on the material (i.e., intermetallic crucible for Al, and graphite for the remaining materials). The evaporation process was performed starting from a base vacuum batter than 10^–7^ mbar.

The proton irradiation plan is summarized in Table 2, where the details of the sessions, in terms of energy, fluence, and flux, are reported for each sample. The energies selected (i.e., 1 keV, 16 keV, 50 keV, 100 keV, 1 MeV, and 10 MeV) were applied to all samples, to test proton implantation profiles of different distributions and densities. The maximum tested fluences are compatible with the worst-case scenario, which is represented by a 15-year mission in the GEO orbit at lower energies and by the SOLO and JUICE mission environments at higher ones. Moreover, fluences at energies ≤50 keV were delivered by using different fluxes, in order to verify if there is any dependency on this parameter. Irradiation experiments were performed at the Ion Beam Center (IBC) of Helmholtz-Zentrum Dresden-Rossendorf (HZDR), Germany. The 1, 4, and 16 keV proton irradiations were performed by using the Danfysik A/S 40 kV ion implanter; the irradiations at 50 and 100 keV were performed with the High Voltage Engineering Europa 500 kV ion implanter, whereas the irradiations at 1 and 10 MeV were performed by using the 3 and 6 MV Tandetron high-energy ion accelerators, respectively. All the implantation sessions were performed at room temperature, while the experimental chamber vacuum was kept at about 10^–7^ mbar (i.e., 10^–5^ Pa). The proton beam was tilted by 7° with respect to the sample-surface normal, which is a typical value adopted in the semiconductor industry for minimizing the channeling effect in the case of materials having crystalline states or a columnar structure. Beam homogeneity was monitored by comparing the measured proton currents at the four corners of a Faraday cup. The ion current was integrated over time in order to control the total fluence. The accuracy associated with the delivered fluences is 5%, whereas that associated with the fluxes is 10%. After irradiation, the samples were stored in the dark in an opaque box at room temperature. For the irradiations at 10 MeV, a period of storage in a safety area was envisaged following the implantation session in order to let any activation of the samples extinguish.

**Table 2:**
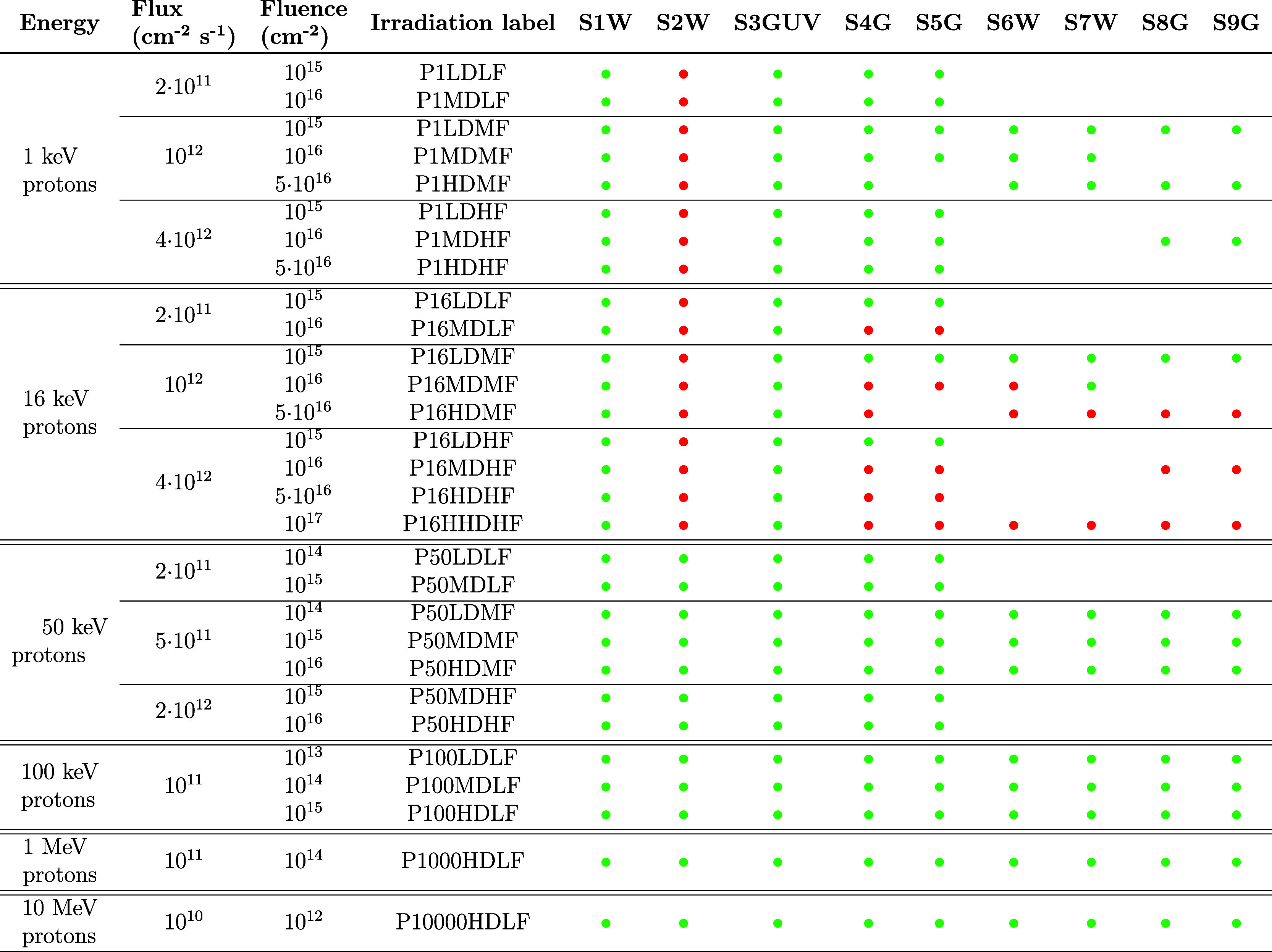
Summary of the Proton Irradiation Plan^a^

a Green dots represent samples that did not experience damage, whereas red dots are used for samples that were affected by the irradiation, as discussed in section 4. Fluences and fluxes accuracy are 5% and 10%, respectively.

Before and after irradiation, the spectral transmittance/reflectance of the samples was measured in the 250–1300 nm wavelength range at a step of Δλ = 1 nm by using a Cary 5000 double-grating spectrophotometer. The absolute reflectance at 7° of normal incidence was measured with the VW-geometry accessory. The measurement accuracy is better than 1%.

For the metallic samples that show performance degradation, the scattering was evaluated by measuring the total integrated scattering (TIS), defined as
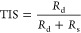
1where *R*_s_ is the specular reflectance and *R*_d_ is the diffuse reflectance. The specular and diffuse reflectance was measured at 3.5° of normal incidence by using the internal diffuse reflectance accessory (IDRA) in the 350–800 nm spectral range. The accuracy of the measured TIS is estimated to be about 4%. The surface morphology was characterized by using an XE-70 Park System atomic force microscope (AFM) operated in noncontact mode.

The crystalline state of the coatings before and after implantation was investigated by grazing-incidence X-ray diffraction (GIXRD). The data were acquired on a Philips MRD diffractometer operated at 40 kV and 40 mA using Cu Kα radiation (λ = 1.540 56 Å). The primary optics consists of a parabolic multilayer mirror collimating and partially removing the contribution of other X-ray lines in the primary beam. Both the sample and the detector (a Xe proportional counter) are mounted on two coaxial high-precision goniometers (accuracy of 0.0001° and repeatability 0.001°).

The structural properties of the proton-implanted films were investigated by cross-sectional bright-field transmission electron microscopy (TEM). These analyses were performed using an image-C_s_-corrected Titan 80-300 microscope (FEI) operated at an accelerating voltage of 300 kV. Classical TEM cross sections of the proton-broad-beam-irradiated samples glued together in face-to-face geometry using G2 epoxy glue (Gatan) were prepared by sawing (Wire Saw WS 22, IBS GmbH), grinding (MetaServ 250, Bühler), polishing (Minimet 1000, Bühler), dimpling (Dimple Grinder 656, Gatan), and Ar^+^ ion milling (Precision Ion Polishing System PIPS 691, Gatan). Cross-sectional preparation of TEM lamellae at particular surface defect structures was done by in situ lift-out using a Helios 5 CX focused ion beam (FIB) device (Thermo Fisher). To protect the defects, a carbon cap layer was deposited beginning with electron-beam-assisted decomposition, followed by Ga-FIB-assisted precursor decomposition. Afterward, the TEM lamella was prepared by using a 30 keV Ga-FIB with adapted currents. Its transfer to a 3-post copper lift-out grid (Omniprobe) was done with an EasyLift EX nanomanipulator (Thermo Fisher). To minimize sidewall damage, Ga ions with only 5 keV energy were used for the final thinning of the TEM lamella to electron transparency.

## Results and Discussion

A preliminary screening of the damaged coatings after each session was carried out by computing a normalized root-mean-square deviation parameter (RMSD), defined as follows:
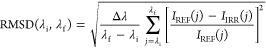
2where *I*_REF_(*j*) is the spectral reflectance or transmittance of the sample before irradiation, *I*_IRR_(j) is the spectral reflectance or transmittance after irradiation, Δλ is the wavelength step used in the measurements, and λ_i_ and λ_f_ define the wavelength range within which the RMSD is computed. Three different wavelength ranges were considered: the UV range (250–400 nm), the VIS range (400–800 nm), and the NIR range (800–1300 nm). When RMSD < 0.01, the irradiation-induced effects are considered negligible, as variations in the reflectance/transmission curves are within measurement uncertainty. In contrast, when RMSD > 0.01, the sample is considered damaged. While some samples show degradation in all three spectral ranges, others are degraded only at short wavelengths, while they preserve their performances at longer ones. As an example, samples S4G and S5G show different damage outcomes when irradiated in the same session with 16 keV protons: while the TiO_2_ single-layer transmittance drops with increasing fluence over the entire UV–VIS–NIR spectrum, the ZrO_2_ single layer changes only in the UV and VIS but not in the NIR (Figures S5 and S6 in Supporting Information). The results of this preliminary analysis are reported in Table 2, where the irradiation session parameters are given, together with the RMSD outcomes for each type of sample: green dots are used to mark the samples with RMSD < 0.01 in all the three ranges, whereas red dots denote a change of the optical performances at least in one of the three spectral regions. As shown, MeV protons never affect the coatings; this result is well in line with those reported in the literature for dielectric materials and dielectric interferential filters^35,48^ and for MgF_2_-protected Al mirrors,^49^ although the fluences used in the previous tests were in general lower. The results can be explained by the fact that given the high energy protons implant into the substrate because of their long stopping range, overtaking the coating with almost no energy release.

Still, irradiations with protons at 50 and 100 keV do not induce appreciable changes in the tested coatings, well in agreement with what was already observed for some dielectrics.^35,36^ Moreover, literature reports test outcomes for ≥60 keV protons irradiated on SiO_2_-protected Al mirrors which show little degradation in the UV reflectance (i.e., <3%), only for fluences up to 10^16^ cm^–2^.^37^ In the present work, the RMSD analysis shows no appreciable degradation for protected aluminum, gold, and silver mirrors irradiated by both 50 and 100 keV, confirming the threshold fluence of 10^16^ cm^–2^ that was found in ref (37).

**Figure 3:**
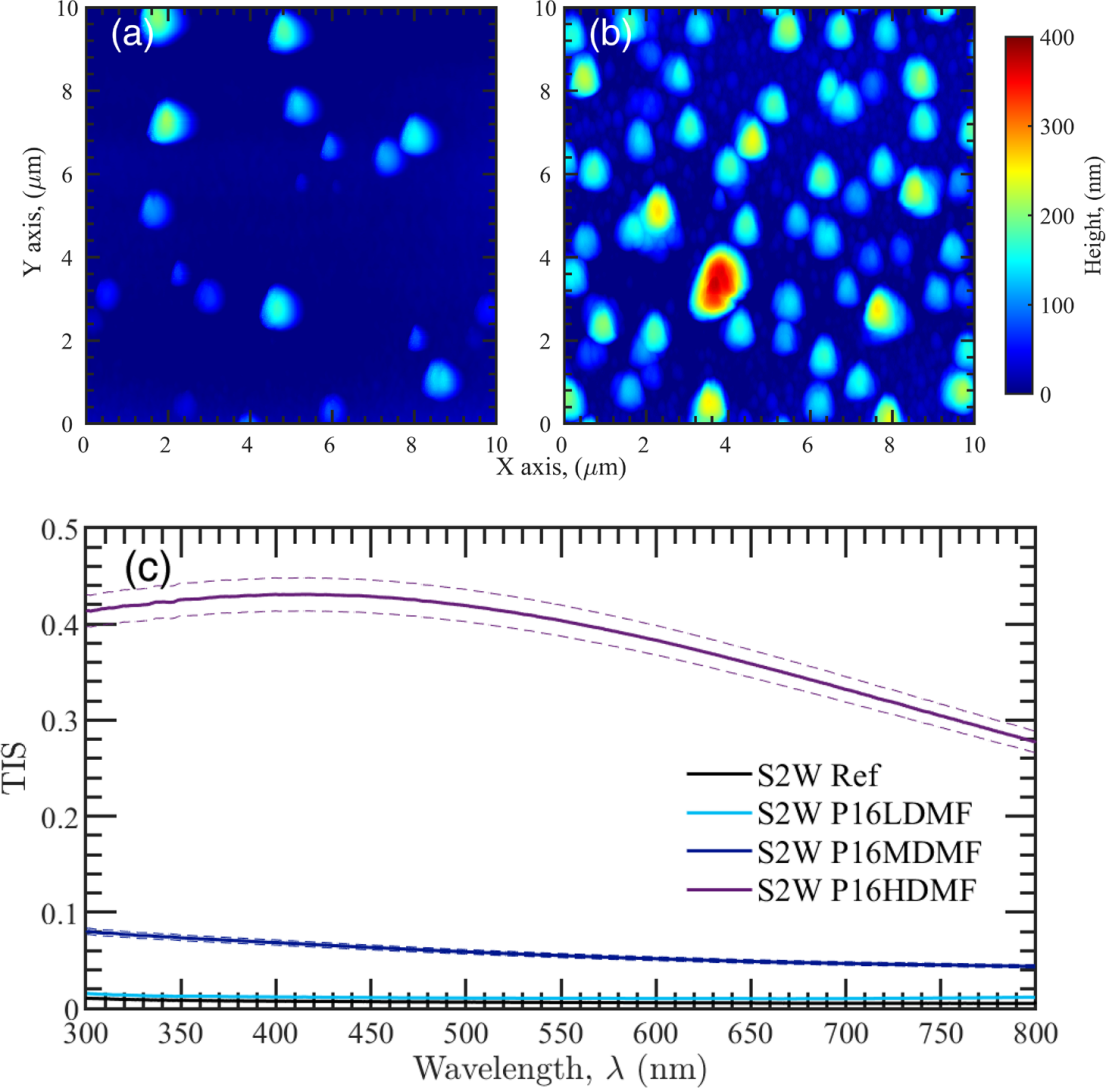
AFM analysis of the bare aluminum (S2W) irradiated with 1 keV protons: (a) sample irradiated with a fluence of 10^16^ cm^–2^ (P1MDMF); (b) sample irradiated with a fluence of 5 × 10^16^ cm^–2^ (P1HDMF). In (c), the TIS analysis of the S2W samples irradiated with proton fluences of 10^15^ cm^–2^ (P1LDMF), 10^16^ cm^–2^ (P1MDMF), and 5 × 10^16^ cm^–2^ (P1HDMF) is reported. Dashed lines indicate the accuracy limits of the measurements.

Considering irradiations at 1 keV energy, we can observe that bare aluminum (S2W) has a reflectance drop already at fluences of the order of 10^15^ cm^–2^ (Figure S2 in Supporting Information). For 1 keV protons, the penetration depth is smaller than in previous cases; the protons implant in the coating close to the surface, and the density of the implanted protons is higher. When low-energy protons are implanted in metals, the damage induced can be mainly attributed to two different processes: nanobubble formation at depths compatible with the protons penetration and local detachment or delamination due to the stress accumulated in the film.^16,17^ The dimensions of the bubbles depend on the material and density profile of the implanted protons, as they are formed through a process of migration and agglomeration of protons. In the case of S2W, the reflectance drop is mainly due to bubble formation close to the surface, inducing surface blistering,^50^ as shown by the AFM analysis reported in Figure 3a,b. A direct consequence of the blistering is an increase in the surface roughness RMS from 3.6 to 47.3 nm and thus in scattering, as revealed by the TIS measurements reported in Figure 3c. The same degradation effect is also found for an energy of 16 keV (Figure S3 in Supporting Information): although the implantation profile is wider, protons still stop in the Al layer, with a density high enough to induce bubble formation. It also has to be noted that Al is a highly reactive material, which makes it prone to oxidation processes due to the residual content of water vapor in the vacuum chamber.

Irradiations with 16 keV protons are proven to be more detrimental than those at 1 keV, as all the tested samples underwent performance degradation with the only exceptions of the bare gold coating (S1W) and the SiO_2_ single layer (S3GUV), which remain stable even up to fluences of the order of 10^17^ cm^–2^. Gold has already been proven to be particularly resistant, as it is capable of withstanding fluences of the order of 10^16^ cm^–2^ when irradiated with He^+^.^17,20,21,51^ In addition, the result obtained for SiO_2_ demonstrates its potential reliability as a protective coating for metallic thin films.

**Figure 4:**
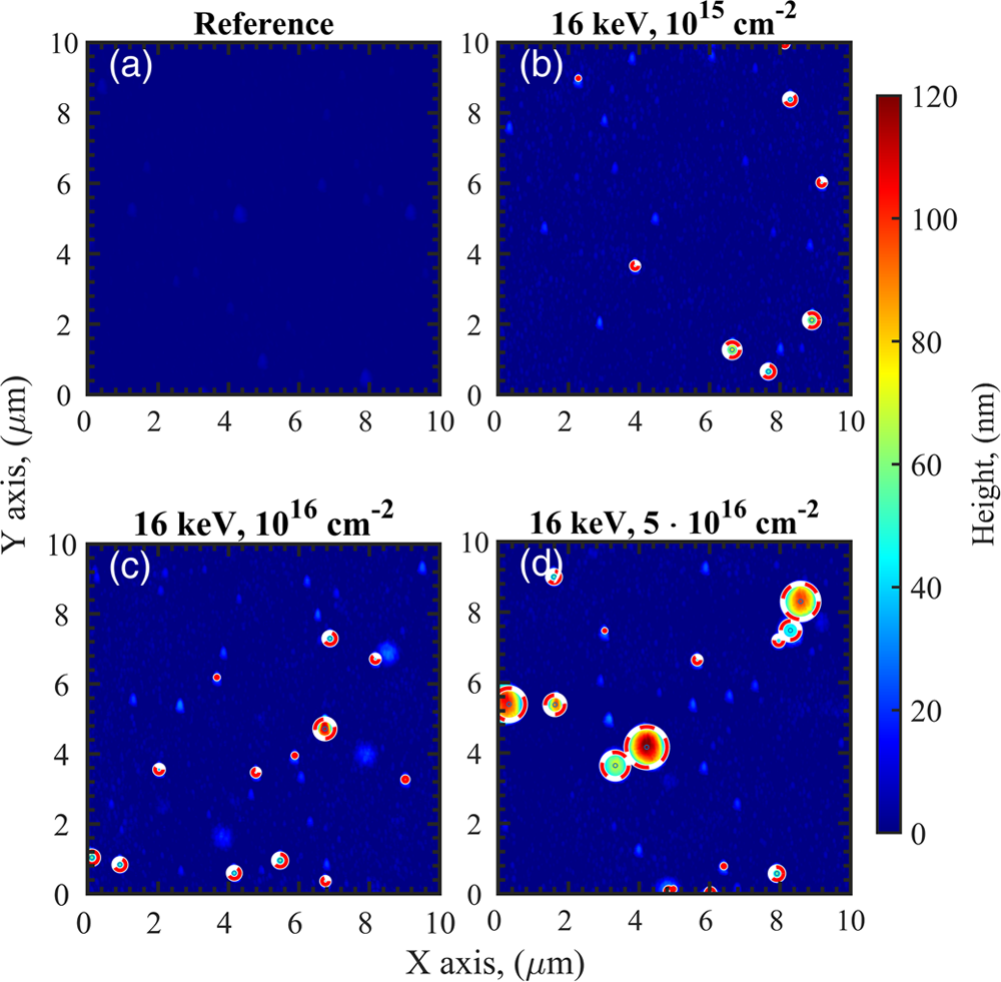
AFM analysis of the Al/SiO_2_ (S6W) samples irradiated with 16 keV protons. (a) Unirradiated reference and samples irradiated with a fluence of 10^15^ cm^–2^ (P16LDMF) (b), 10^16^ cm^–2^ (P16MDMF) (c), and 5 × 10^15^ cm^–2^ (P16HDMF) (d). In the AFM images, bubbles with a height of ≥30 nm are marked with red-white circles.

**Figure 5:**
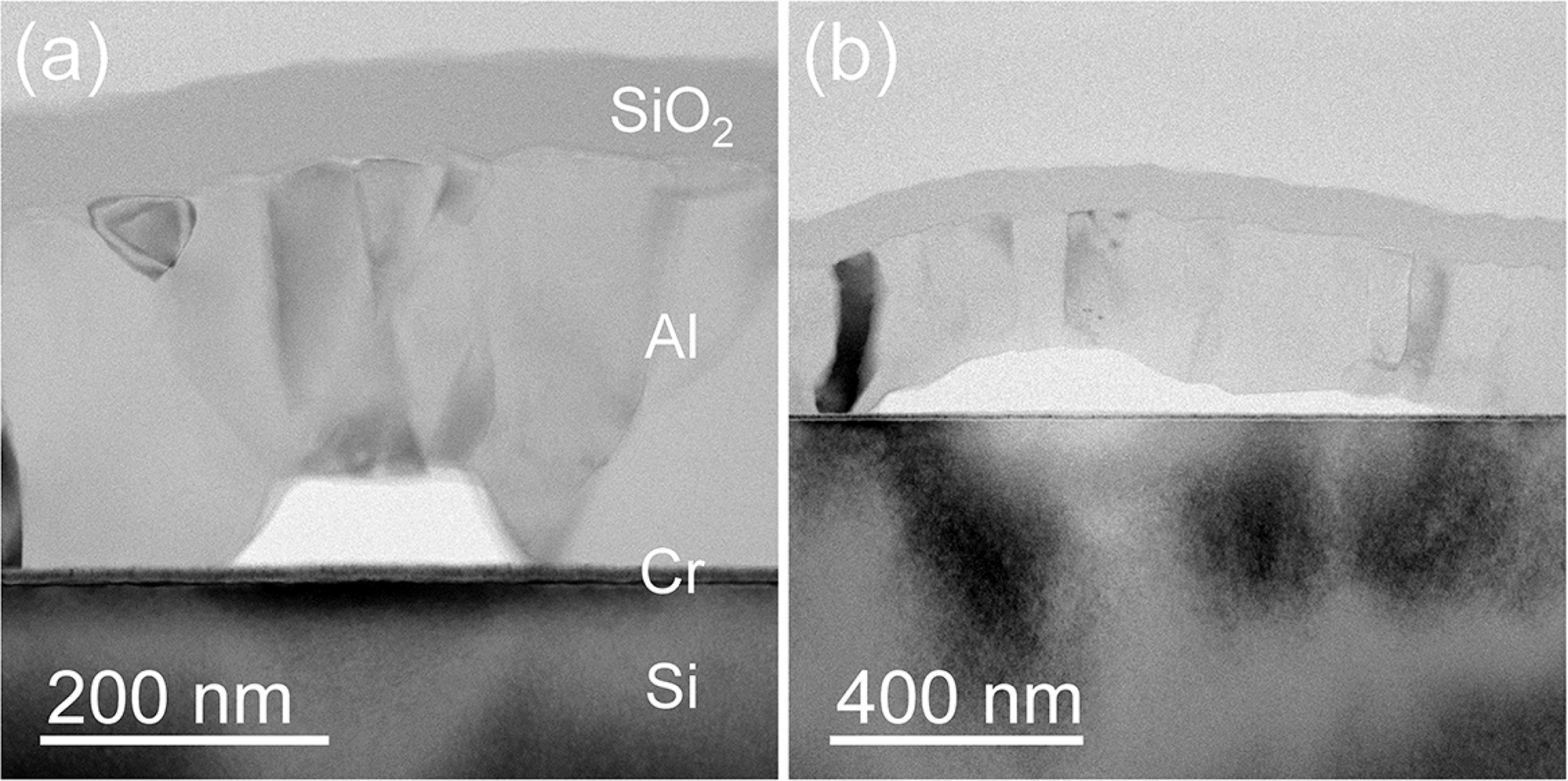
Cross-sectional bright-field TEM images of the SiO_2_-protected Al mirror (S6W) irradiated with a proton fluence of 5 × 10^16^ cm^–2^ at 16 keV (P16HDMF). (a) Formation of a small agglomeration at the Al/Cr interface, having a diameter of ≃200 nm and a height of ≃60 nm. Such a bubble induces a film deformation which produces a surface swelling of ≃0.4 μm in diameter and height similar to that of the bubble. (b) Formation of a large bubble and the consequent delamination (i.e., right part of the void) at the Al/Cr interface.

**Figure 6:**
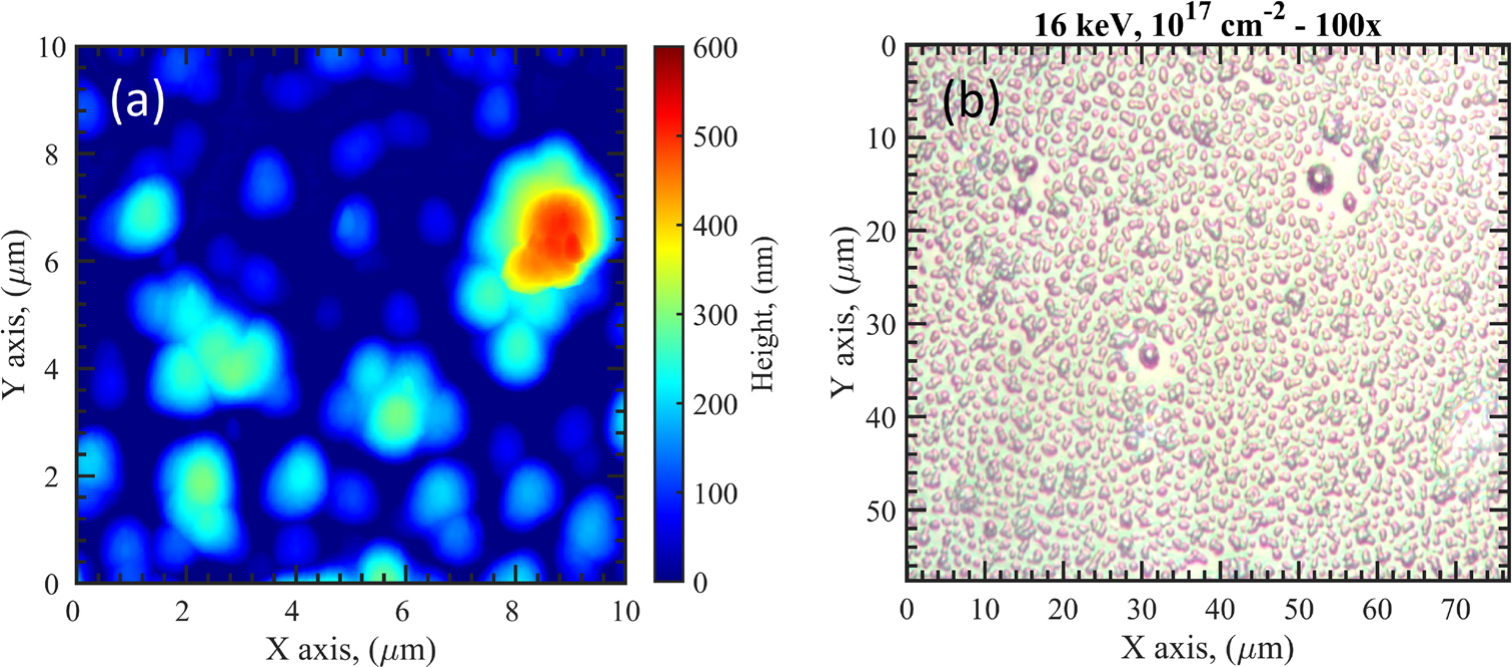
(a) AFM and (b) optical microscopy images of the Al/SiO_2_ (S6W) sample irradiated with 16 keV protons and a fluence of 10^17^ cm^–2^ (P16HHDHF).

**Figure 7:**
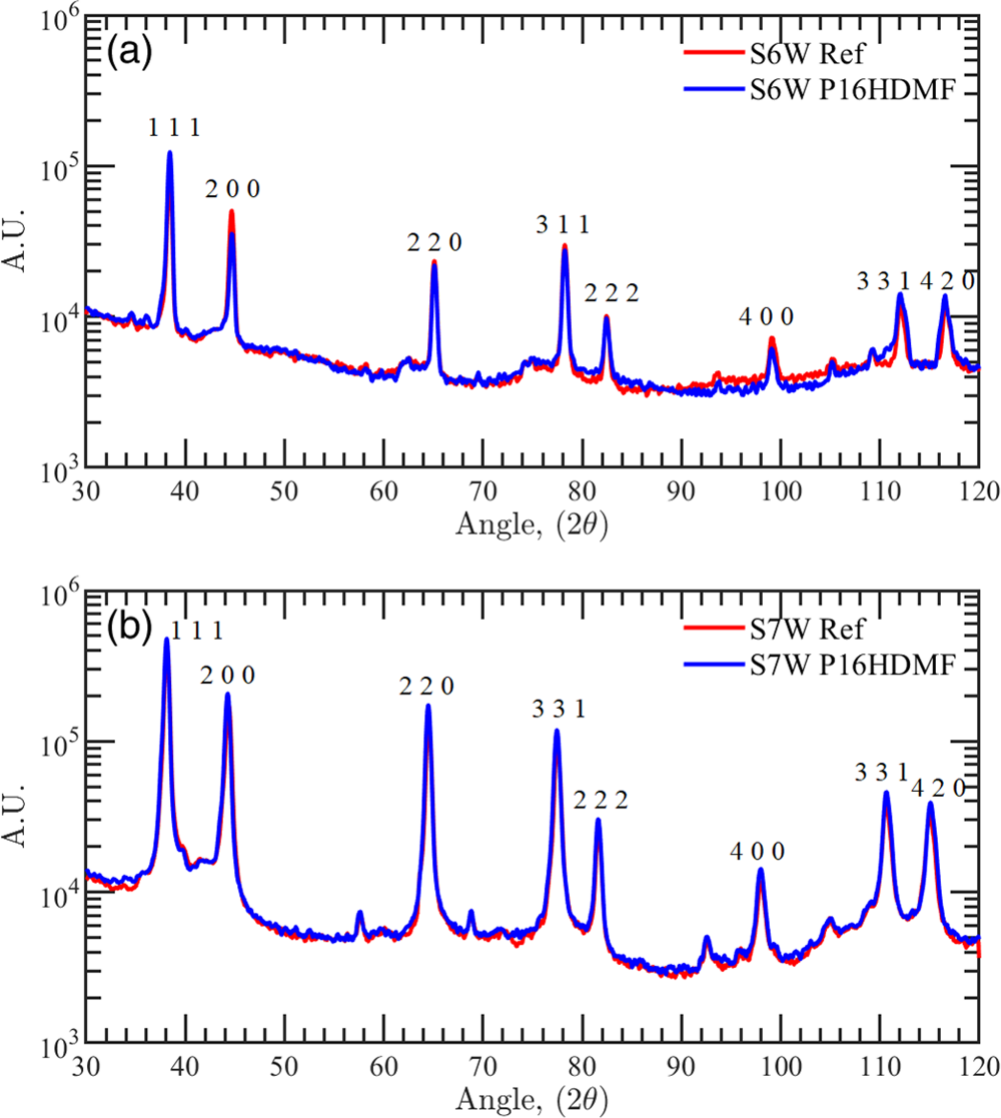
X-ray diffractogram for the (a) SiO_2_/Al sample (S6W) and (b) SiO_2_/Ag sample (S57W). Red curves report the XRD for the unirradiated samples (Ref), whereas the blue curves report the XRD for the irradiated samples with 16 keV protons with a fluence of 5 × 10^16^ cm^–2^.

However, while the SiO_2_-protected aluminum mirror (S6W) remains stable for 1 keV proton irradiation, as the particles implant in the dielectric, for the 16 keV proton case, it is stable only up to a fluence of ≃10^15^ cm^–2^ (Figure S7 in Supporting Information). In fact, 16 keV protons can reach the Al film, and the damage is again due to bubble formation, as revealed by AFM analysis reported in Figure 4. In particular, the unimplanted S6W reference sample does not reveal any relevant features (the surface roughness of this sample is *R*_*q*_ ≃ 3.7 nm), with the exception of some sporadic lumps with a height always ≤10 nm (Figure 4a). On the other hand, the surface morphology changes in the sample irradiated with a fluence of 10^15^ cm^–2^ (Figure 4b), as small bubbles with a diameter of ≤0.5 μm and heights up to 60–70 nm begin to appear. At a fluence of 10^16^ cm^–2^, the density of the surface bubbles increases further: while diameters are still of the order of 0.2–0.7 μm, heights can reach values beyond 100 nm (Figure 4c). Moreover, in the same image, additional formations appear, which consist of swellings of ≃20 nm height and ≃1 μm diameter. Such structures become more evident for the 5 × 10^16^ cm^–2^ fluence, as blubbles of ≥1–2 μm diameter and ≥100 nm height are present on the surface (Figure 4d). Cross-sectional TEM analysis shows that the bubbles observed by AFM originate from delamination at the interface between the substrate and the metallic layer; such delaminations originate from the stress induced in the film by the implanted protons. Figure 5 reports two examples of such delaminations occurring at a fluence of 5 × 10^16^ cm^–2^: a smaller bubble is shown in Figure 5a, while a larger one with a diameter of several hundreds of nanometers is present in Figure 5b; the sizes of these structures are compatible with the small and large formations observed on the surface by AFM (Figure 4d). As expected, the sample is fully damaged at a fluence of 10^17^ cm^–2^, as the surface is completely covered with bubbles, which can reach even diameters of ≃5 μm and heights ≥500 nm; the blistered surface is also visible with an optical microscope (Figure 6). Additional GIXRD investigations on the S6W samples before and after proton irradiation did not show any relevant change in the polycrystalline structure of the Al film, which remains in its cubic phase (see Figure 7a).

**Figure 8:**
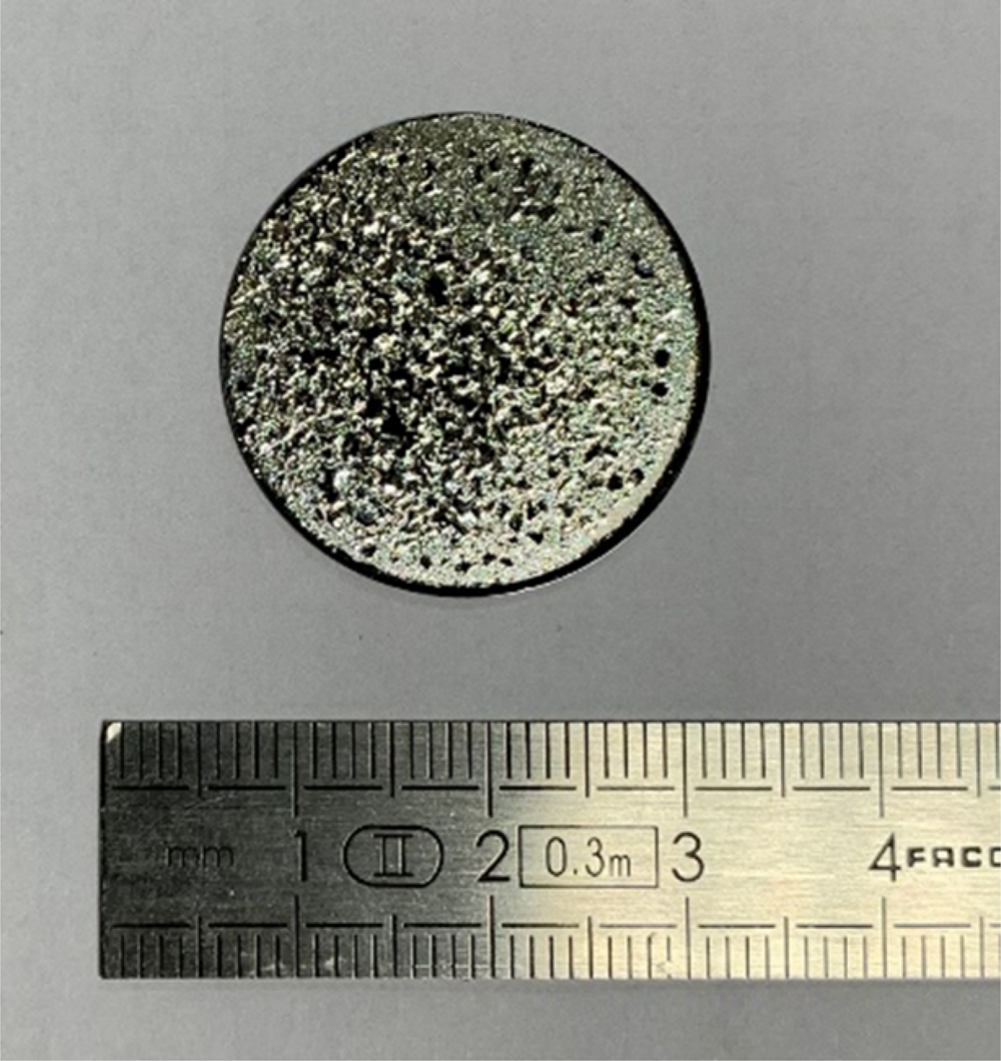
Picture of sample S7W after 16 keV proton irradiation with a fluence of 10^17^ cm^–2^.

The SiO_2_-protected silver (S7W) is stable for fluences up to ≃10^16^ cm^–2^ in the visible and at least up to 5 × 10^16^ cm^–2^ in the NIR, proving to be more resistant than the most commonly used protected aluminum mirror. Moreover, S7W remains stable not only in the NIR, where it is commonly used, but also in the UV (Figure S8 in Supporting Information). However, for a fluence of the order of ≃10^17^ cm^–2^, the sample is fully damaged, showing blistering and consequent coating delamination (Figure 8). Also in this case, the GIXRD measurements did not show any changes in the crystalline structure, confirming that the main cause of degradation is the formation of bubbles in the metal layer (see Figure 7b).

Regarding dielectric thin films, the optical transmittance curves of TiO_2_ (S4G) and ZrO_2_ (S5G) single layers reveal that the two materials degrade differently (see Figures S5 and S6 in Supporting Information). While both samples are stable in the UV and VIS range when irradiated with a 16 keV proton beam at a fluence up to ≃10^15^ cm^–2^, above this value, TiO_2_ (S4G) exhibits a consistent degradation in the whole spectral range, whereas ZrO_2_ (S5G) degrades to a lesser extent, primarily in the UV and visible range. This behavior persists even at the maximum fluence of 10^17^ cm^–2^; in this case, the roughness of S4G as measured by AFM increases from 0.63 nm up to 4 nm, mainly due to the appearance of small surface structures of 7–8 nm maximum height and a lateral size of the order of <100 nm. In the case of S5G, the roughness is not so pronounced, as it increases from 1.07 up to 2.15 nm, with no formation of particular structures on the surface.

**Figure 9:**
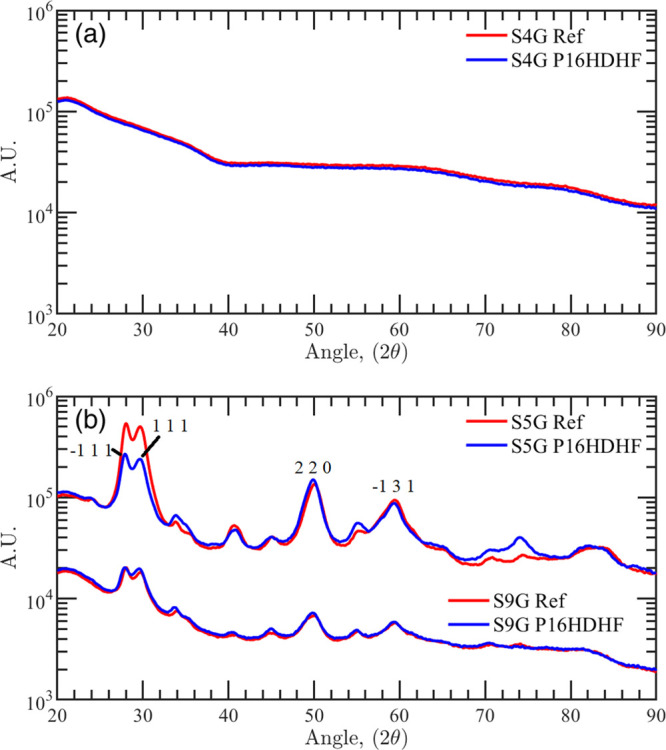
X-ray diffractogram for the (a) TiO_2_ single-layer sample (S4G) and (b) ZrO_2_-based samples (S5G and S9G). Red curves report the XRD for the unirradiated samples (Ref), whereas the blue curves report the XRD for the irradiated samples with 16 keV protons with a fluence of 5 × 10^16^ cm^–2^.

**Figure 10:**
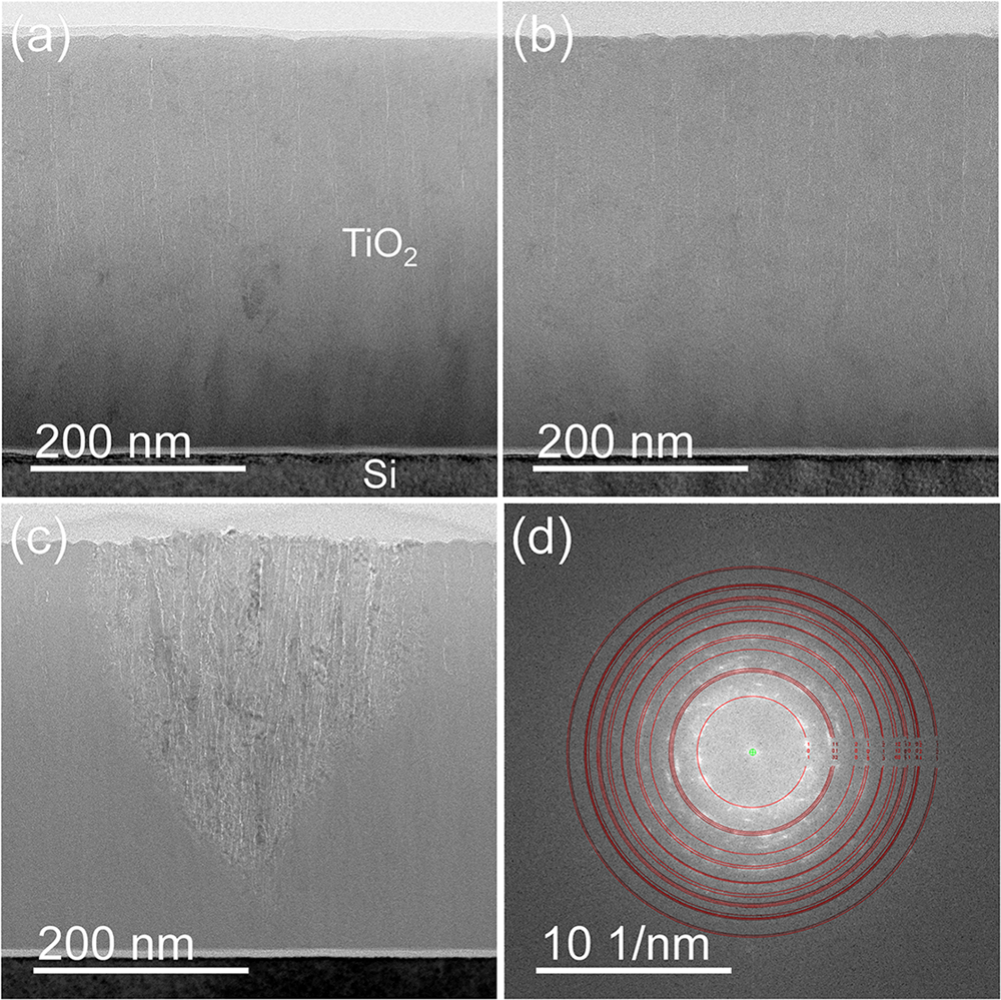
Slightly defocused cross-sectional bright-field TEM images of the TiO_2_ single layer deposited on a silicon wafer (S4W): (a) reference, (b) irradiated with 16 keV protons and a fluence of 10^16^ cm^–2^ (P16MDMF), (c) irradiated with 16 keV protons and a fluence of 10^17^ cm^–2^ (P16HHDHF), (d) description of the FFT of a high-resolution TEM image from the highly crystalline cone region shown in (c) with the TiO_2_ anatase phase.

GIXRD analysis of the bare TiO_2_ indicates that the film is amorphous both prior to and after all proton irradiation sessions (see Figure 9a). According to TEM analysis, the TiO_2_ layer is mainly amorphous but contains small crystalline inclusions for all samples irradiated with a fluence up to 1 × 10^16^ cm^–2^ (see the slight diffraction contrast variations within the TiO_2_ layer region in Figure 10a and b). However, TEM images of the sample irradiated with a fluence of 10^17^ cm^–2^ (P16HHDHF) show the formation of many cone-shaped regions, with the cone base on the sample surface and its apex close to the film/substrate interface. Within the cone, strong crystallization occurred (Figure 10c). Describing the fast Fourier transform (FFT) of a high-resolution image obtained from this highly crystalline cone region based on the assumption of an unaltered TiO_2_ composition, a prevalent anatase phase can be concluded (Figure 10d), while small rutile contributions might be present, too. Furthermore, it was verified by scanning electron microscopy that such cone-shaped regions exactly coincide with small structures present on the sample surface, previously observed via AFM.

The GIXRD data of the ZrO_2_ single layer show both the zirconia monoclinic crystalline phase (i.e., −111 and −131 peaks) and the tetrahedral crystalline phase (i.e., 111 and 220 peaks) (see Figure 9b). This crystalline state is slightly modified by proton irradiation, as demonstrated by the diffraction patterns obtained for the sample irradiated with a fluence of 5 × 10^16^ cm^–2^ and reported in Figure 9b. The decrease in the relative intensity of the diffraction peaks −111 and 111 with respect to the other peaks can be reasonably attributed to a reduction of the crystalline state of the zirconia, which produces the change observed in the optical transmission.

**Figure 11:**
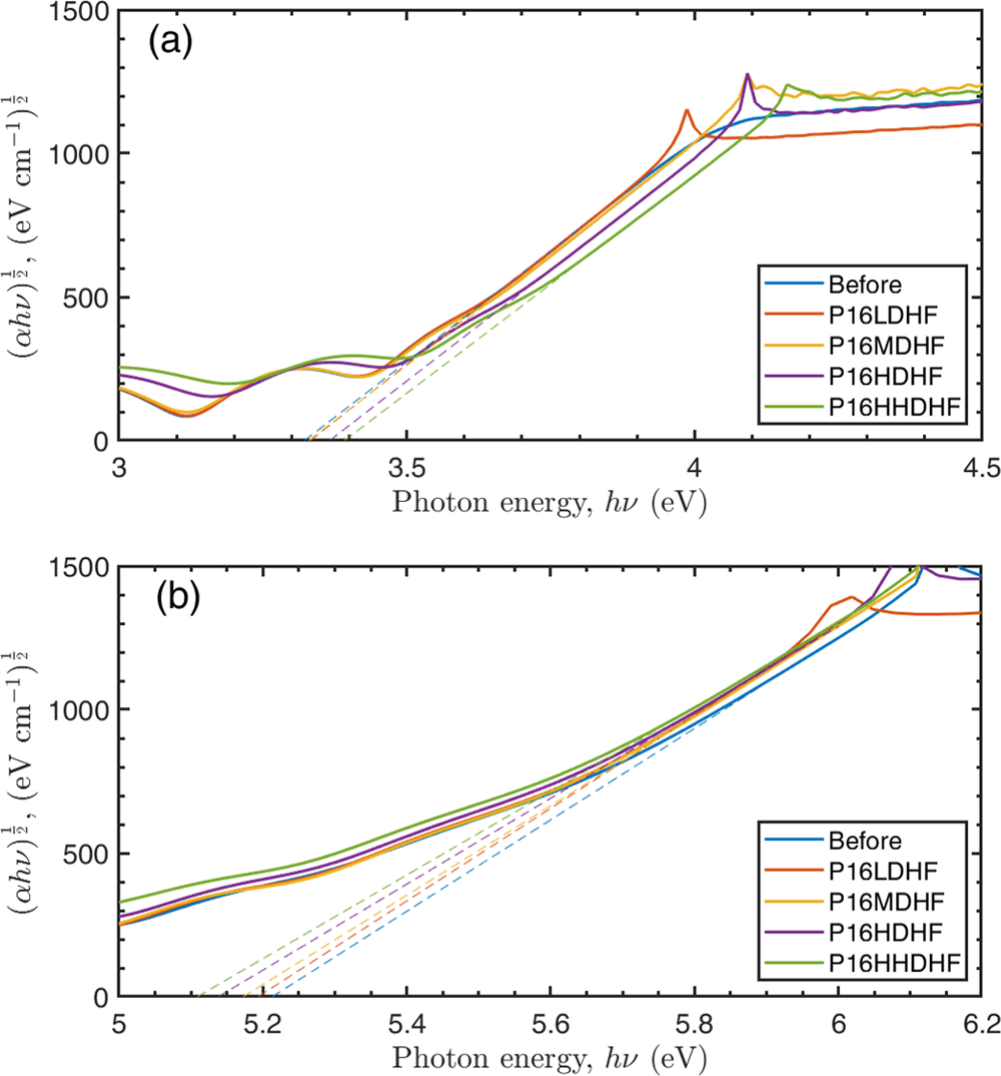
Tauc plot for the TiO_2_ single-layer sample (S4G) (a) and for the ZrO_2_ single-layer sample (S5G) (b) irradiated with 16 keV protons with HF flux.

For the single-layer samples S4G and S5G, the optical band gap as a function of the proton fluence has been evaluated. According to interband absorption theory, optical absorption strength depends on the difference between the photon energy and the optical band gap *E*_g_ as^52^
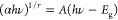
3where *h* is Planck’s constant, ν is the photon’s frequency, α is the absorption coefficient, *E*_g_ is the optical band gap, and *A* is a constant which does not depend on the photon energy *h*ν. The value of the exponent denotes the nature of the electronic transition: *r* = 1/2 for direct allowed transitions, *r* = 3/2 for direct forbidden transitions, *r* = 2 for indirect allowed transitions, and *r* = 3 for indirect forbidden transitions. The best fits for the samples under investigation were obtained considering an indirect allowed transition and then *r* = 2. At shorter wavelengths, close to the optical absorption edge, the material absorption dominates the scattering losses, allowing to estimate of the absorption coefficient as α = −ln(*T*)/*d*, where *d* is the film thickness and *T* is the transmittance measured via the spectrophotometer.^52,53^ Therefore, the optical band gap can be evaluated by extrapolating the straight line part of the curve  with the energy axes *h*ν i.e.,  The indirect optical band gap of TiO_2_ and ZrO_2_ thin films was evaluated for the samples irradiated with 16 keV protons; Figure 11 reports only the optical band gap obtained for the set of samples irradiated with HF, since similar results were obtained for the samples irradiated with the lower fluxes. Thus, the induced changes on the optical band gap seem to be dependent on only the fluence.

Before the proton irradiation, the optical energy gap of the TiO_2_ single-layer sample was estimated to be 3.32 ± 0.01 eV. Such value agrees with values reported in the literature, where the optical band gap of the amorphous TiO_2_ is collocated in the 3.3–3.4 eV range.^54,55^ The proton irradiation seems to not affect the optical energy gap for fluences below 10^16^ cm^–2^ (i.e., 3.33 ± 0.01 eV for both fluences). However, a clear blue shift is observed for the fluences at 5 × 10^16^ cm^–2^ (HD) and 10^17^ cm^–2^ (HHD), being 3.37 ± 0.01 eV and 3.39 ± 0.01 eV, respectively. Such a blue shift is probably due to the formation of the local highly crystalline regions observed in the TEM images (Figure 10c), which appears for high fluences. ZrO_2_ single-layer samples exhibited the exactly opposite behavior after irradiation, their optical band gap decreases as the fluence increases (Figure 11b). The unirradiated sample shows a band gap of 5.21 ± 0.01 eV. For the irradiated samples, the band gap decreases to 5.19 ± 0.01, 5.17 ± 0.01, 5.13 ± 0.01, and 5.11 ± 0.01 eV as the proton fluence increases from LD to HHD. It is reasonable to attribute this optical band gap red shift to the modification in the initial crystalline state of the ZrO_2_, as already revealed by the GIXRD analysis.

Considering the bilayer samples of SiO_2_/TiO_2_ and SiO_2_/ZrO_2_, the analysis does not reveal any significant change prior to and after irradiation. This result is not surprising, considering that in both samples approximately half of the impinging protons implant into the SiO_2_ layer, and the majority of the proton beam’s energy is deposited in this topmost layer. Indeed, the SiO_2_ layer puts in place its protective function effectively, substantially mitigating the effects observed in the layers susceptible to proton irradiation (i.e., titania or zirconia). As a result, even with the highest fluences considered in this study, the crystalline changes and formations of crystallization areas discussed above are significantly less pronounced and they are not clearly detectable via TEM or GIXRD analysis. However, their occurrence is suggested by the degradation of the transmittance curves on the irradiated samples, which is less than that observed on the unprotected samples.

In the discussion of the causes of degradation, chemical composition changes have not been considered, even though they cannot a priori be excluded. However, the increase of temperature in samples during irradiation is not meaningful, and the selected samples are known as being stable over time. Moreover, since the formation of chemical compounds at interfaces affects only a few nanometer thicknesses, their effect on transmittance and reflectance is evident in the vacuum ultraviolet and soft-X-rays regimes but not in the spectral ranges considered in the present paper.

Apart from S1W and S3GUV, which are stable for any proton energy and irradiation fluence considered in the present study, and S2W, which is damaged after any of the low-energy proton irradiation sessions, the safety fluence value *F*_s_ for each type of sample is summarized in Table 3. It represents the maximum fluence at which the sample does not show any significant change in reflectance or transmittance performance in each of the three spectral ranges (UV, VIS, and NIR). In the case of samples that do not nominally perform in the UV and thus are not used in such a spectral range, the maximum fluence is not reported.

**Table 3:** Summary of the Safety Fluence Values *F*_s_ Retrieved for Samples Irradiated with 16 keV Protons^a^

sample label	materials in structure	spectral range	*F*_s_, maximum fluence without *R*/*T* drop [cm^–2^]		
			LF	MF	HF
S4G	TiO_2_	VIS	10^15^	10^15^	10^15^
		NIR	10^15^	10^15^	10^15^
S5G	ZrO_2_	UV	10^15^	10^15^	10^15^
		VIS	10^15^	10^15^	10^15^
		NIR	10^16^	5 × 10^16^	10^17^
S6W	SiO_2_/Al	UV		10^15^	
		VIS		10^15^	
		NIR		10^15^	
S7W	SiO_2_/Ag	VIS		10^16^	
		NIR		5 × 10^16^	
S8G	SiO_2_/TiO_2_	VIS		10^15^	*
		NIR		10^15^	*
S9G	SiO_2_/ZrO_2_	UV		10^15^	*
		VIS		10^15^	*
		NIR		5 × 10^16^	10^17^

a The symbol * indicates the case in which a reflectance *R* or transmittance *T* drop was present already at the minimum tested fluence of 10^16^ cm^–2^; in such cases, the data available do not allow indication of a safety fluence.

## Conclusions

This work presents the results of a systematic study of low-energy proton irradiation carried out on optical coating samples (monolayers and bilayers) made with various materials. The dielectric materials investigated are SiO_2_, TiO_2_, and ZrO_2_, which are typically employed in antireflection coatings, interferential filters, and protective layers of metallic films. Building blocks made by SiO_2_/TiO_2_ and SiO_2_/ZrO_2_ have also been tested to verify the potential damages induced at the interface between the two materials. Three different metals used in reflective coatings were also considered: Al for the UV–VIS range, Ag for the VIS–IR range, and Au for the IR range. To avoid oxidation, Ag has been protected by SiO_2_, while Al was realized with and without a SiO_2_ capping layer.

Various samples of the same coating type were irradiated with protons at different energies and fluences. Single-layers of Au (S1W) and SiO_2_ (S3GUV) never degrade; this result confirms that gold is a very stable and effective metal coating for the selected spectral ranges, and SiO_2_ can be properly used as the topmost protective layer for the coatings intended to work in harsh space environments. For proton energies equal to or greater than 50 keV, all the samples studied in this work never degrade. For proton energy at 1 keV, only bare Al (S2W) shows a degradation; in general, bare Al is very critical at any energy below 50 keV and it should never be used without a protecting layer. For all the other samples (from S4G to S9G), potential damage appears with 16 keV proton irradiation; this result is not surprising, as the samples were intentionally designed to induce the maximum stress on the samples when exposed to proton irradiation at this energy. For the energy of 16 keV, *F*_s_, the maximum fluence at which the sample does not show any significant change in reflectance or transmittance, is reported for each of the three spectral ranges (UV, VIS, and NIR). In the case of the TiO_2_ single layer (S4G) and ZrO_2_ single layer (S5G), some experiments in which the same fluence was supplied but varying the flux were also carried out. For such tests, the same *F*_s_ was determined for both materials, demonstrating that, at least within this experiment, flux does not play a role. However, it must be remembered that fluxes were already selected to be as low as possible and in agreement with values suggested in previous tests, as reported in the Introduction. Morphological and structural analysis atomic force microscopy (AFM), transmission electron microscopy (TEM), and grazing incidence X-ray diffraction (GIXRD) were used to investigate the root causes of the damage observed on the samples irradiated with 16 keV protons. The damage mechanism observed in the samples is dependent on the material. In metal-based samples SiO_2_/Al (S6W) and SiO_2_/Ag (S7W), the degradation is due to large bubble formation close to the interface between metal and substrate/other layers, which occurs when protons overcome the protection layer and implant into the metal layer. In some cases, such bubbles can achieve large dimensions, leading to the local delamination of the coating. The results indicate that sizing the thickness of the protective layer is fundamental to correctly protecting the layer underneath, and this should be defined by simulation of the proton density profile implanted in the coating. In dielectric materials, the optical performance changes are driven by the modification of the crystalline state of the materials, which can occur widely within the film or only be localized in specific areas.

The results obtained in this first systematic study provide indications about safety fluences to which coatings can be exposed to. The results obtained should be taken into consideration for the selection, optimization, and qualification of materials and structures in optical systems for future space missions. Despite the high number of samples considered in this study, some materials and combinations have not been included and should be investigated in the future; for instance, MgF_2_ is widely used as a protective layer on both Al mirrors for vacuum UV and Au for IR applications, while other dielectrics, such as Al_2_O_3_ and Ta_2_O_5_ can be employed in antireflection coatings structure. Moreover, as the substrate can affect the coating growth characteristics, it would be interesting to systematically investigate the low energy (<16 keV) proton irradiation on samples deposited on different glass and ceramic materials. Most of the coatings considered are usually deposited with evaporation techniques; however, some of them can also be realized by a magnetron-sputtering deposition process. In the future, it would also be interesting to verify how the deposition technique can affect the stability of coatings in harsh space environments.

## Supporting Information Available

Spectrophotometric measurements of all samples irradiated with 16 keV protons and of S2W samples irradiated with 1 keV protons (PDF)
