# Supplementary material for: Optical Thin Films in Space Environment: Investigation of Proton Irradiation Damage
Source: ACS Appl Mater Interfaces. 2024 Jul 9;16(29):38645–57. doi: 10.1021/acsami.4c03362 (PMC11284746; doi:10.1021/acsami.4c03362)
Supplement: Supplementary file 1 — am4c03362_si_001.pdf [file am4c03362_si_001.pdf]

Supporting Information

Optical thin films in space environment: investigation of proton irradiation damage

Alain J. Corso [*,†] Marta Padovani [‡] Giovanni Santi [¶] René Hübner [§] Ulrich Kentsch [§] Marco Bazzan [‖] and Maria G. Pelizzo [*,‡,†,¶]

†*Consiglio Nazionale delle Ricerche - Istituto di Fotonica e Nanotecnologie (CNR-IFN), via Trasea, 7, 35131 Padova, Italy*

‡*Università di Padova, Dipartimento di Ingegneria dell'Informazione, via Gradenigo 6B, 35131 Padova, Italy*

¶*Università di Padova, Centro di Ateneo di Studi e Attività Spaziali (CISAS), via Venezia, 15, 35131 Padova, Italy*

§*Helmholtz-Zentrum Dresden-Rossendorf, Institute of Ion Beam Physics and Materials Research, Bautzner Landstr. 400, 01328 Dresden, Germany*

‖*Università di Padova, Dipartimento di Fisica e Astronomia, via Marzolo 8, 35131 Padova, Italy*

E-mail: alainjody.corso@cnr.it; mariaguglielmina.pelizzo@unipd.it

Spectrophotometric measurements of the samples

Spectrophotometric measurements of all samples irradiated with 16 keV protons and of samples S2W irradiated with 1 keV protons.

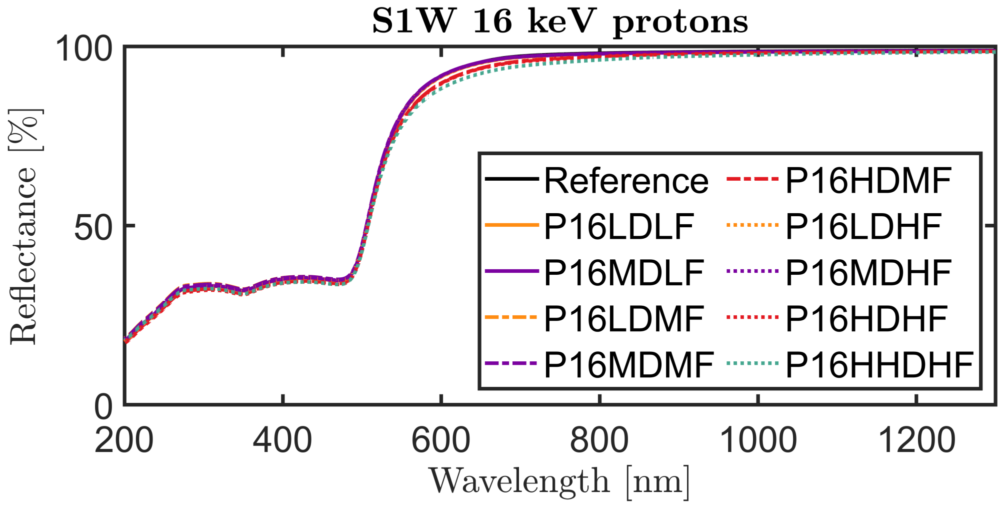

Figure S1: Gold single-layer, spectrophotometric measurements after 16-keV proton irradiation.

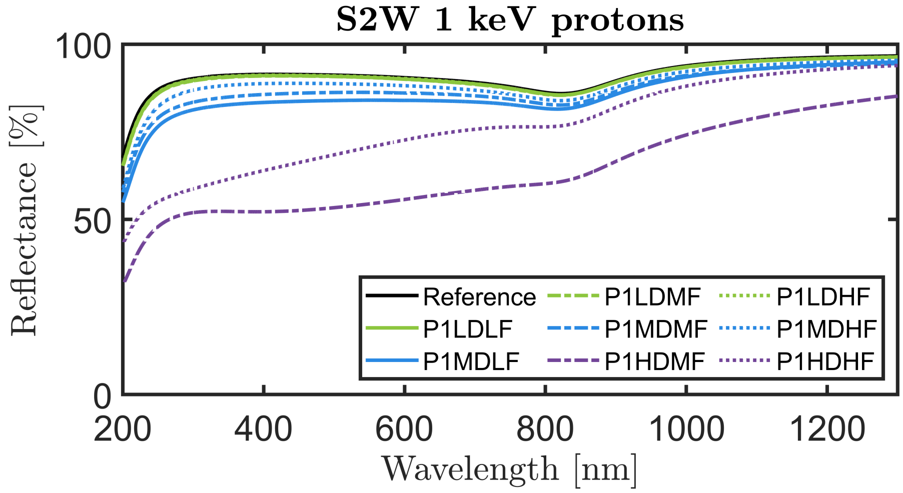

Figure S2: Aluminum single-layer spectrophotometric measurements after 1-keV proton irradiation.

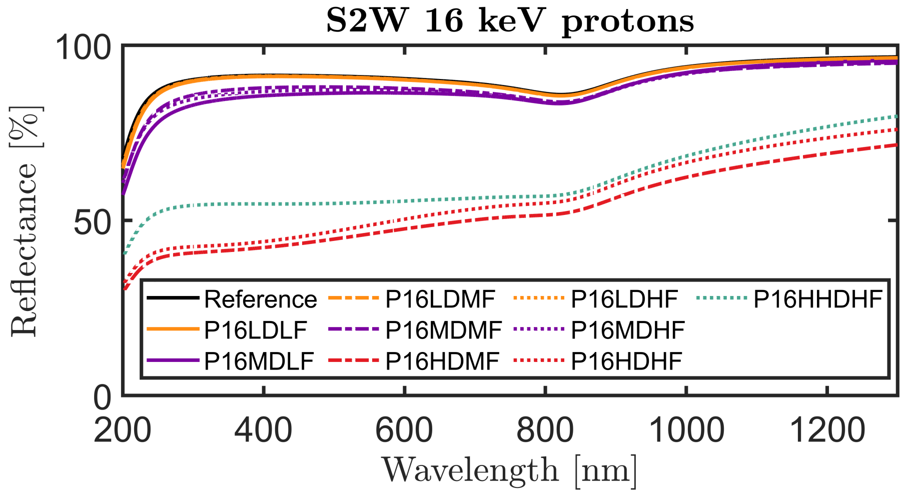

Figure S3: Aluminum single-layer spectrophotometric measurements after 16-keV proton irradiation.

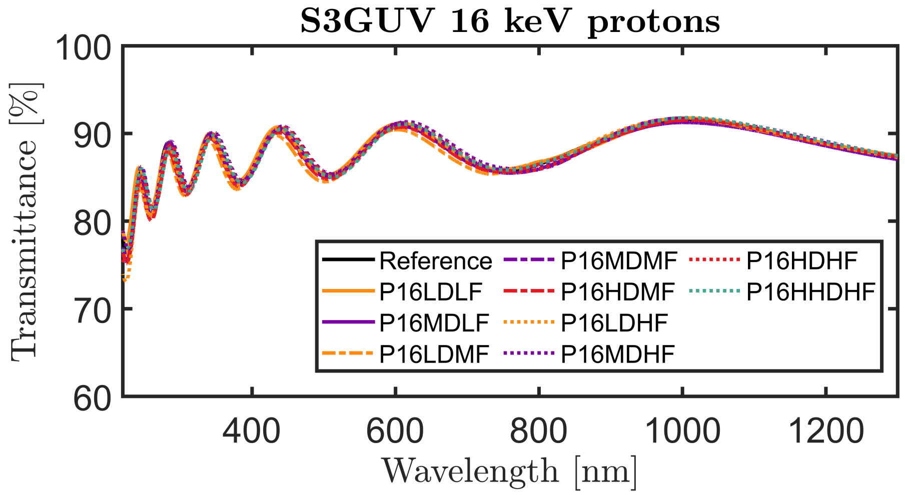

Figure S4: Silicon dioxide single-layer spectrophotometric measurements after 16-keV proton irradiation.

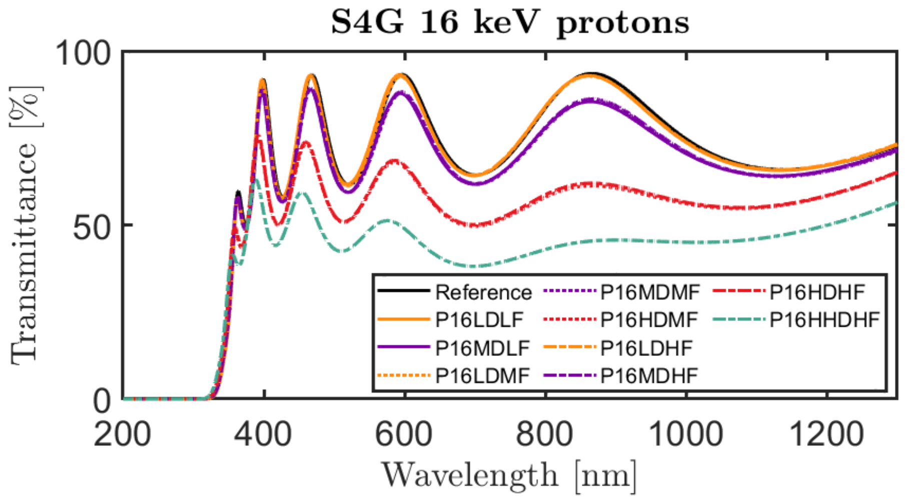

Figure S5: TiO_2 single layer (S4G) spectrophotometric measurements after 16 keV proton irradiation.

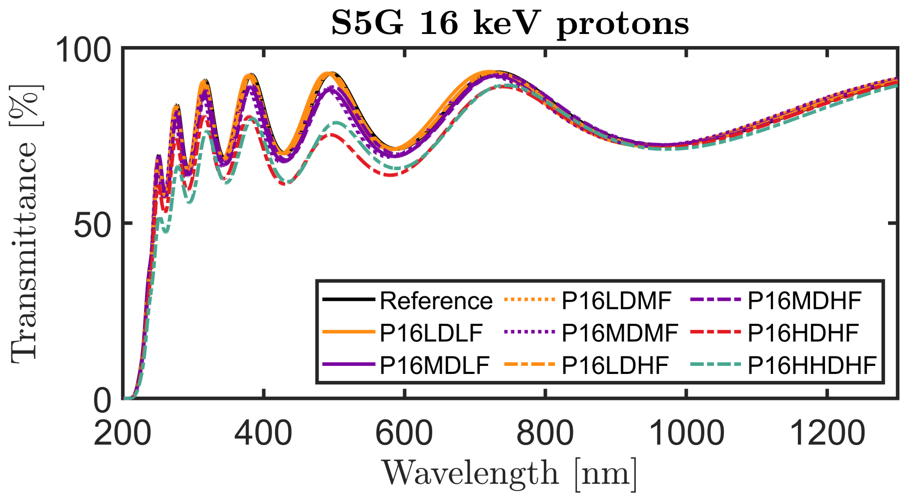

Figure S6: ZrO_2 single-layer spectrophotometric measurements after 16-keV proton irradiation.

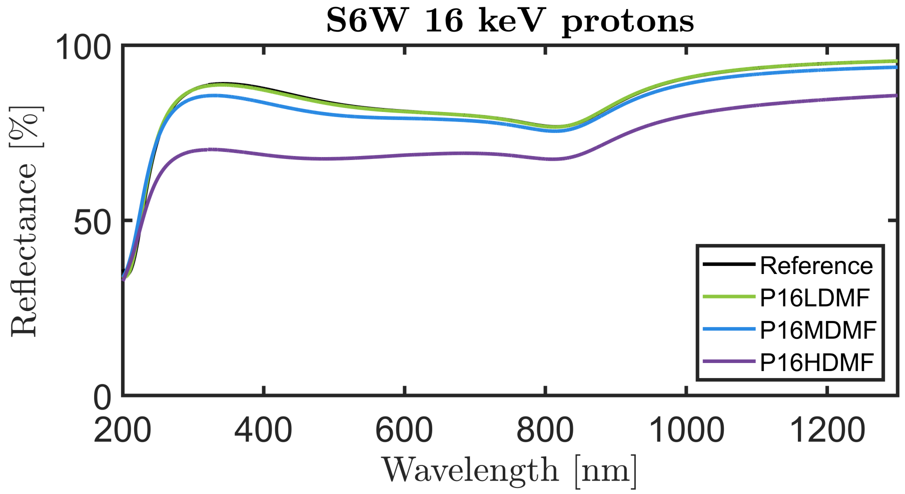

Figure S7: Al/SiO_2 (S6W) bilayer spectrophotometric measurements after 16-keV proton irradiation.

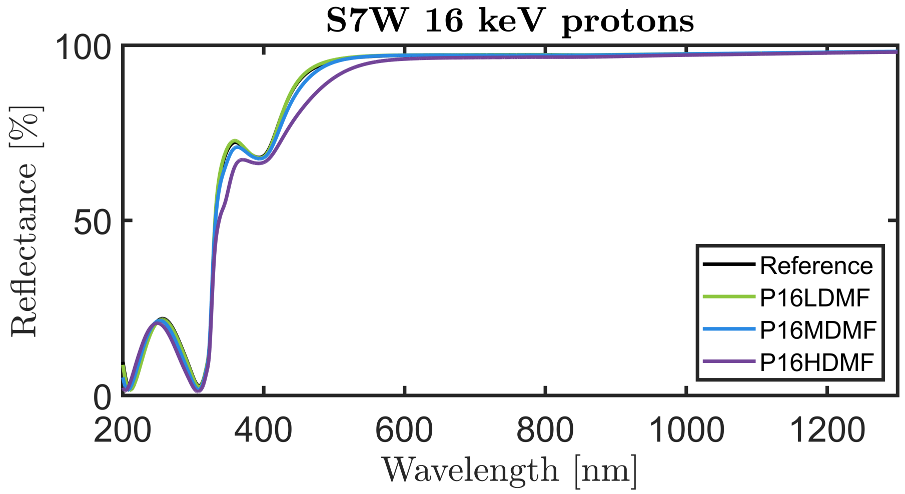

Figure S8: Ag/SiO_2 (S7W) bilayer spectrophotometric measurements after 16-keV proton irradiation.

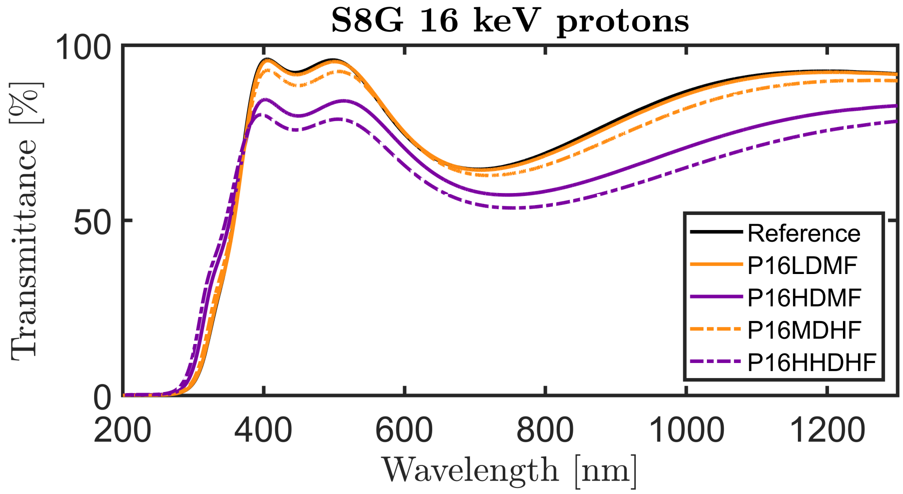

Figure S9: SiO_2/TiO_2 (S8W) bilayer spectrophotometric measurements after 16-keV proton irradiation.

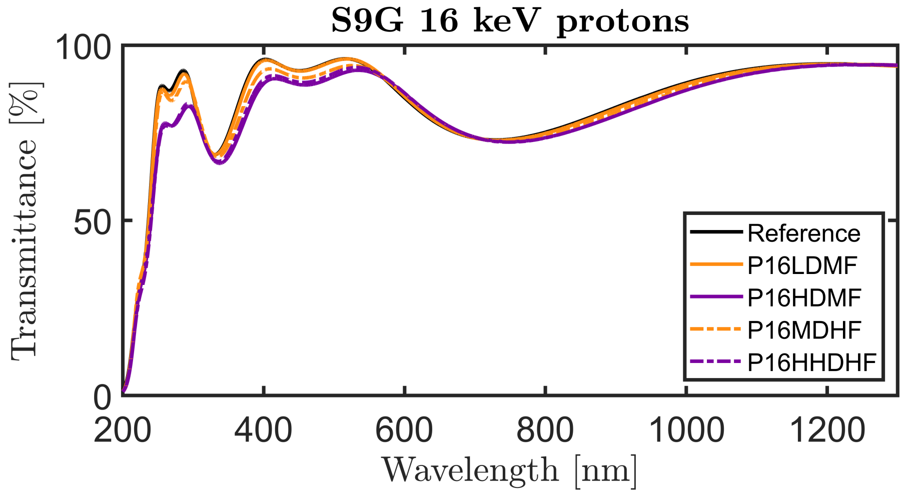

Figure S10: SiO_2/ZrO_2 (S9G) bilayer spectrophotometric measurements after 16-keV proton irradiation.
